# Orthopedic Management of Radial Tunnel Syndrome: A Diagnostic and Treatment Dilemma

**DOI:** 10.5152/eurasianjmed.2023.22274

**Published:** 2023-02-01

**Authors:** Muhammed Çağatay Engin, Mehmet Demirel, Abdullah Kahraman, Ömer Ayık

**Affiliations:** 1 Department of Orthopaedics and Traumatology, Atatürk University Faculty of Medicine, Erzurum, Turkey; 2 Department of Orthopedics and Traumatology, İstanbul University İstanbul Faculty of Medicine, İstanbul, Turkey

**Keywords:** Hand and upper extremity, orthopedics and traumatology, decompression, surgical, radial neuropathy/therapy, nerve compression syndromes/therapy

## Abstract

**Objective:**

The first-line treatment for radial tunnel syndrome is conservative despite limited evidence concerning its efficiency. Surgical release is indicated if nonsurgical measures fail. Radial tunnel syndrome cases may be misdiagnosed as the more common lateral epicondylitis, and misdiagnosing radial tunnel syndrome causes wrong treatment and, thus, the perpetuation or increase of the pain. Although radial tunnel syndrome is a rare disorder, such cases can be encountered in tertiary hand surgery centers. This study aimed to present our experience in diagnosing and managing patients with radial tunnel syndrome.

**Material and Methods:**

Eighteen patients (7 male, 11 female; mean age = 41.5 years, age range = 22-61) in whom radial tunnel syndrome was diagnosed and treated at a single tertiary care center were retrospectively reviewed and included. Previous diagnoses (wrong diagnosis, delayed diagnosis, missed diagnosis, and other), previous treatments for such diagnoses, and their results before presenting to our institution were recorded. The shortened disabilities of the arm, shoulder, and hand questionnaire score and visual analog scale score were recorded before the surgery and at the final follow-up appointment.

**Results:**

All the patients included in the study underwent steroid injections. Eleven patients (11/18, 61%) benefited from steroid injection and conservative treatment. The remaining 7 patients refractory to conservative treatment were offered surgical treatment. Of these, 6 patients accepted surgery while 1 did not accept it. In all patients, the mean visual analog scale score significantly improved from 6.38 (range: 5-8) to 2.1 (range: 0-7) (*P* < .001). The mean quick-disabilities of the arm, shoulder, and hand questionnaire scores were significantly improved from 43.4 (range: 31.8-52.5) preoperatively to 8.7 (range: 0-45.5) at the final follow-up (*P* < .001). In the surgical treatment group, the mean visual analog scale score significantly improved from 6.1 (range: 5-7) to 1.2 (range: 0-4) (*P* < .001). The mean quick-disabilities of the arm, shoulder, and hand questionnaire scores were significantly improved from 37.4 (range: 31.2-45.5) preoperatively to 4.7 (range: 0-13.6) at the final follow-up (*P* < .001).

**Conclusion:**

Our experience has shown that satisfactory results can be obtained by surgical treatment for patients with radial tunnel syndrome refractory to nonsurgical treatment whose diagnosis is confirmed by a thorough physical examination.

**Main Points**

- The first-line treatment for radial tunnel syndrome (RTS) is conservative despite limited evidence concerning its efficiency.
- Surgical release is indicated if nonsurgical measures fail.
- Radial tunnel syndrome cases may be misdiagnosed as the more common lateral epicondylitis, and misdiagnosing RTS causes wrong treatment and, thus, the perpetuation or increase of the pain. This study aimed to present our experience in diagnosing and managing patients with RTS.
- Our experience has shown that satisfactory results can be obtained by surgical treatment for patients with RTS refractory to nonsurgical treatment whose diagnosis is confirmed by a thorough physical examination.

## Introduction

Radial tunnel syndrome (RTS) is caused by entrapment of the posterior interosseous nerve (PIN), the motor branch of the radial nerve, in the proximal forearm. In this extremely rare syndrome (0.03%), nerve conduction tests, radiological studies, and pathophysiologic findings are typically negative, without motor and sensory dysfunction signs. The major manifestation of RTS is clinical, and thus physical examination plays a pivotal role in diagnosing such a rare disorder. The typical physical finding is the pain with palpation at 4 to 5 cm distal to the lateral epicondyle over the supinator muscle mass.^1-3^

The first-line treatment for RTS is nonsurgical management despite limited evidence concerning its efficiency. Surgical release is needed when nonsurgical methods fail. Radial tunnel syndrome cases may be misdiagnosed as the more common lateral epicondylitis in a similar location (1%-3%).^4^ Misdiagnosing patients with RTS leads to the wrong treatment and, thus, the perpetuation or increase of the pain. Although RTS is a rare disorder, such cases can be encountered in tertiary hand surgery centers. This study aimed to present our experience in diagnosing and managing such cases.

## Materials and Methods

The medical records of 20 patients in whom RTS was diagnosed and treated at a single tertiary care center between 2017 and 2020 were retrospectively reviewed. Inclusion criteria were (1) a diagnosis of RTS, (2) at least 12 months of follow-up, (3) complete clinical medical records, and (4) being willing to participate in the study. Exclusion criteria included (1) concomitant entrapment neuropathy in the same extremity, (2) RTS cases accompanied by lateral epicondylitis, (3) concomitant systemic peripheral neuropathy, (4) RTS cases secondary to trauma or tumor, (4) lost to follow-up, and (5) being unwilling to participate in the study.

All the patients were evaluated based on the above eligibility criteria. After excluding 2 patients (1 patient was lost to follow-up and 1 patient had concomitant lateral epicondylitis), the remaining 18 patients (7 male, 11 female) were enrolled in the study and invited to a final follow-up examination. The Clinical Researches Ethics Committee of Atatürk University, School of Medicine approved this study before data collection, and all participants gave informed consent. (Decision No: B.30.2.ATA.0.01.00/302)

## Outcome Measures

Demographic and clinical data were obtained from the hospital’s electronic database and the patient’s medical records, including age at the time of surgery, gender, presentation of symptoms and signs on admission, follow-up period, time to return to work, and occupational history. Previous diagnoses (wrong diagnosis, delayed diagnosis, missed diagnosis, and other), previous treatments for such diagnoses, and their results before presenting to our institution were recorded.

Clinical examinations used for the diagnostics were a meticulous medical history, pain palpation tests, and pain provocation tests. The provocative tests included pain with resisted extension of the middle finger with the forearm pronated and the elbow extended and pain with resisted forearm supination with the elbow fully extended. Conventional x-rays, magnetic resonance imaging (MRI), and electromyography (EMG) were then done in all cases to exclude the differential diagnosis of RTS. In the differentiation of RTS from lateral epicondylitis, the localization of pain in lateral epicondylitis is more proximal than the localization of RTS, in the area above the lateral epicondyle. It also differs in provocative tests.

The shortened disabilities of the arm, shoulder, and hand questionnaire (QuickDASH) score^5^ and visual analog scale (VAS) score^6^ were measured immediately before the surgery and at the final follow-up appointment. The intensity of hand use during everyday activities before decompression surgery was classified as none, light, moderate, or vigorous. The level of patient satisfaction with the decompression surgery was rated as very disappointed, disappointed, somewhat satisfied, satisfied, or very satisfied. Final follow-up assessments were conducted by an independent hand surgeon who did not participate in any of the operations.

### Diagnostic Protocol

In all patients with a suspected diagnosis of RTS, a local injection was done. The patient’s forearm was first supinated, and the most painful area along the radial nerve course was identified with palpation on the dorsum of the forearm, starting from just proximal to the lateral epicondyle. Then, to confirm the diagnosis of RTS, a single local corticosteroid injection (1 mL of betamethasone) in combination with a local anesthetic (3 mL of bupivacaine hydrochloride) was applied to the area of maximal tenderness with a 22 G needle. After injection, the patient was asked if pain persisted with palpation of the tender area. If pain was relieved, the diagnosis of RTS was established, and the following non-surgical (conservative) treatment was initiated: (1) immobilization with a wrist splint to inhibit the forearm motion, (2) activity modifications such as avoiding prolonged forearm pronation, wrist flexion, or elbow extension, and (3) non-steroidal anti-inflammatory medications for 3 to 6 months. Patients were then followed- up at 3 weeks, 6 weeks, 3 months, and 6 months. All patients underwent only a single injection therapy; if the pain persisted, a second attempt was not conducted.

## Surgical Treatment Protocol

Surgical exploration and decompression were performed on patients whose symptoms did not respond to the nonsurgical treatment methods. The decompression was applied to the radial nerve and either of its branches: the superficial branch of the radial nerve and the PIN (the deep motor branch of the radial nerve). 

**Figure 1.**
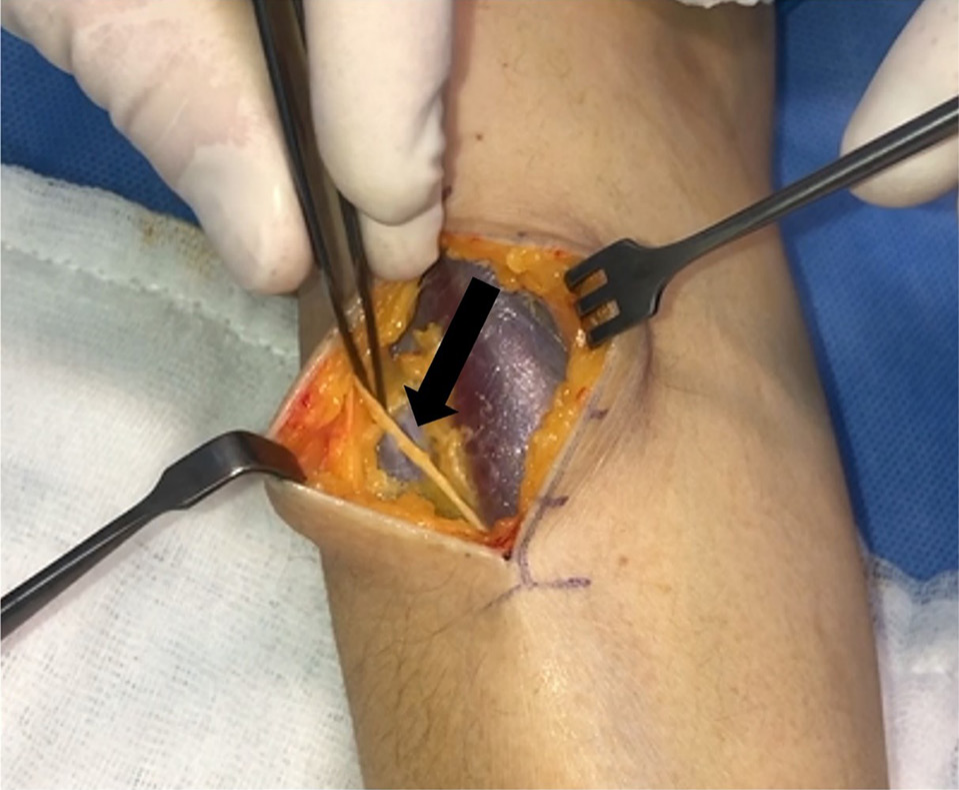
Cutaneous nerve (black arrow) is identified and protected with skin retractor.

**Figure 2.**
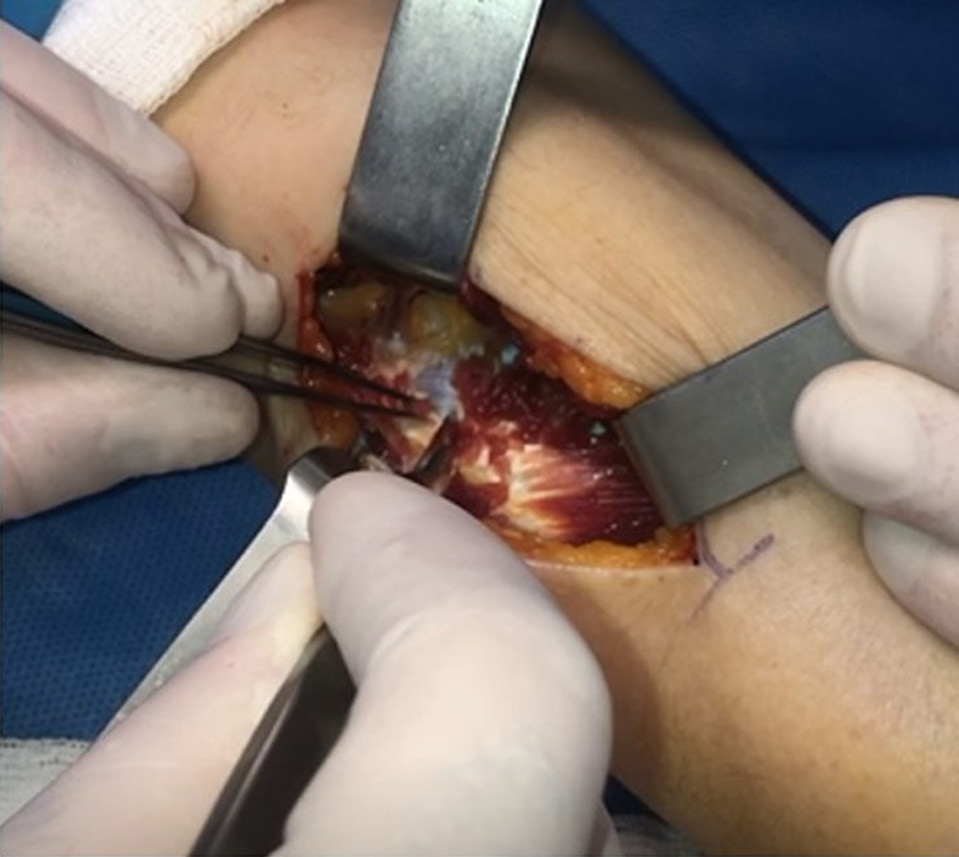
The supinator muscle is exposed. Proximally, the fascia and muscle are started to be loosened from the body.

**Figure 3.**
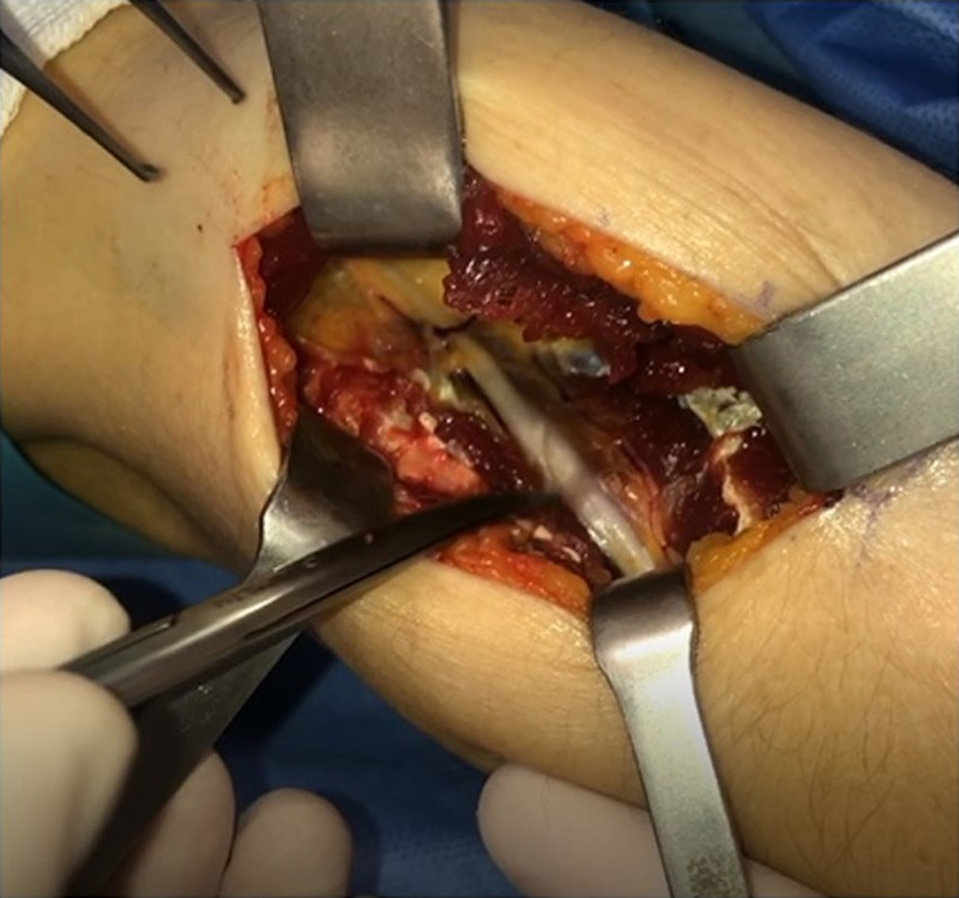
Posterior interosseous nerve is seen after supinator muscle release.

Each operation was performed by the same surgeon, who was specialized in upper limb surgery. Under regional anesthesia, the patient was placed in the supine position, and a pneumatic tourniquet was applied to the upper arm. The elbow was placed on an arm table with the forearm in pronation. A longitudinal skin incision about 8-10 cm was made 2 cm distal to the lateral epicondyle and extended along the midline of the wrist. The posterior cutaneous nerve of the forearm was identified and protected (Figure 1). Using the dorsal approach, the interval between the brachioradialis and extensor carpi radialis longus was developed, and then the supinator muscle was exposed between the 2 muscles by blunt dissection (Figure 2). At the proximal side of the supinator muscle, the Frohse arcade was identified as a tendinous band, and at just proximal to this arcade, the radial recurrent blood vessels, known as leash of Henry, was ligated. After that, while protecting the PIN, the Frohse arcade, the fascia of the superficial supinator muscle, and the muscle trunk were released and the nerve was completely decompressed (Figure 3). Finally, the tourniquet was deflated, and bleeding control was done. The drain was not used, and the skin and subcutaneous tissue were closed.

### Postoperative Management

Postoperative splinting was not used. The dressing was changed every 3-4 days. A home exercise program supported by rehabilitation, early active elbow movements within the limits of pain, and nerve gliding exercises were started immediately postoperatively. The patients were restricted from doing heavy work for up to postoperative 8 weeks, except for daily housework. After 8 weeks postoperatively, all patients were allowed to do upper limb daily activities without any restrictions.

### Statistical Analysis

All statistical analyses were performed with Statistical Package for Social Sciences software version 22.0. (IBM SPSS Corp.; Armonk, NY, USA). Nonparametric paired comparisons were made using the Wilcoxon signed-rank test. A *P*-value < .05 was considered significant.

## Results

There were 18 patients in the study (7 males; 11 females) with a mean age of 41.5 (range = 22-61) years. There were 9 right and 9 left hands, with the dominant hand involved in 10 cases. The mean time to onset of symptoms was 10.5 (range = 5 to 20) months, and the mean follow-up after surgical treatment was 20 (range = 12 to 32) months. The intensity of hand use during everyday activities before decompression surgery was determined as none in 3 patients, light in 4, moderate in 6, and vigorous in 5 (Table 1).

Out of total 18 patients, 6 (33%) were misdiagnosed with lateral epicondylitis, who underwent local corticosteroid injection and lateral epicondylitis band. Of these 6 patients, 3 stated their pain became worse with the treatment.[Table t2-eajm-55-1-59] [Table t3-eajm-55-1-59]

All the patients included in the study underwent steroid injections. Eleven patients (11/18, 61%) benefited from steroid injection and conservative treatment. The remaining 7 patients refractory to conservative treatment were offered surgical treatment. Of these, 6 patients accepted surgery while 1 did not accept it.

In clinical examination, palpation of the tender area elicited pain in all patients. None of the patients had motor and sensory deficits. In provocation tests, middle finger extension against resistance was positive in 8 patients, and resisted supination test was positive in 10 patients. Initial radiographs and EMG were negative in all patients. On MRI, 2 patients displayed denervation edema in the supinator muscle.

While pain on palpation resolved in 11 patients in the main group treated conservatively, 7 patients continued. Resisted long finger extension test was positive in 3 patients, and resisted supination test was positive in 5 patients. When the satisfaction of the treatment results was questioned, in the conservative group, which was our first-step treatment, 9 patients were very satisfied, while 2 patients were satisfied, 2 patients were disappointed, and 5 patients were very disappointed. The mean VAS score significantly improved from 6.38 (range: 5-8) to 2.1 (range: 0-7) (*P* < .001). The mean QuickDASH was significantly improved from 43.4 (range: 31.8-52.5) preoperatively to 8.7 (range: 0-45.5) at the final follow-up (*P* < .001) (Table 2). In the patient group who underwent surgery, the mean VAS score significantly improved from 6.1 (range: 5-7) to 1.2 (range: 0-4) (*P* < .001). The mean QuickDASH was significantly improved from 37.4 (range: 31.2-45.5) preoperatively to 4.7 (range: 0-13.6) at the final follow-up (*P* < .001). Pain with palpation and positiveness for resisted long finger extension test, and resisted supination test continued in just 1 patient who was disappointed (Table 3).

Of the 6 patients who were dissatisfied and accepted our surgical recommendation, 4 patients were very satisfied, 1 patient was satisfied, and 1 patient was disappointed after surgery. A second operation was re-offered to the dissatisfied patient, but the patient refused. While the perioperative PIN image of 3 of the 6 operated patients was normal, the PIN of 3 patients was flattening at the level of the swelling arc proximal to the Froshe arch. There was no patient who could not return to or change his profession due to pain, including the patient whose complaint continued. While no complication was observed in any of the patients after the injection, a local wound problem was observed in 1 patient who underwent surgery, which healed with oral antibiotic therapy and dressing.

## Discussion

There are limited number of studies with a small number of cases on RTS, which presents with pain and difficulty in forearm functions caused by pain. Since there are very few studies on the effectiveness of conservative treatment, it has been shown to be low-effective until recently, but in recent years, very successful results have been reported, especially with single steroid injection.^7,8^ In our study, we observed positive results in 11 patients (61%) with single steroid injection to the most sensitive point. With the direct injection into the arcade of Froshe under USG, García et al^9^ observed more positive results (over 90%) and reduced the risk of complications such as partial nerve damage compared to the injection without USG.

Surgical satisfaction has been reported as high as 67%-92% in studies,^10^ but some studies are not so optimistic.^11^ Another controversial point is the region where the nerve is trapped. Possible regions are the capsular tissue of the radiocapitellar joint, the radial recurrent blood vessels (Henry), the sharp medial edge of the extensor carpi radialis brevis, the proximal aponeurotic edge of the supinator (also known as the arcade of Frohse), and the supinator muscle itself.^3^ In our study, we released the arcade of Frohse and supinator muscle, which is the most common entrapment area of the nerve, and we ligate the leash of Henry, which is one of the potential entrapment areas. We observed positive results with surgery (5 out of 6, 83%). There is no comparison between approaches in this entrapment neuropathy. We prefer the approach between the brachioradial and extensor carpi radialis longus, which we think is easy dissection.

Radial tunnel syndrome is a syndrome whose etiology has not been fully understood. It is a dynamic process rather than a static process, and during repetitive pronation and supination of the wrist and forearm, endoneural inflammation, edema, and demyelination may develop in the nerve due to a dynamic compression process.^12^ Perhaps they benefit from steroid injection for this inflammatory cause. However, the long-term effect of this benefit and the long-term recurrence rates of pain have not been studied yet. It has been reported that the incidence of RTS is higher in patients with male gender and intense shoulder rotation during the day.^13^ In our series, different from the literature, women (61%) were in the majority. And these patients were housewives who stated that they frequently did daily household chores such as cleaning and cooking, which required forearm rotation. However, in our series, there were 7 (3 none + 4 mild) (39%) patients with occupational necessities and no suspicious findings in etiology.

Radial tunnel syndrome is a rare syndrome that does not have any electrodiagnostic and pathophysiologic findings and may be difficult for the clinician to initially diagnose, is often confused with LE(Lateral Epycondilitis) due to its regional proximity. Six of the patients in our series were misdiagnosed, and the pain of 3 patients increased even more which we thought was due to the compressive effect of the LE bands, which were preferred in the previous treatment of the patients, on the proximal forearm. The localization difference between RTS and LE is easily understood even with only a careful physical examination. If there is no healing despite the treatment applied in LE patients, it is necessary to be careful in terms of RTS. In some patients, it can be seen together with LE. In the literature, this association has been shown to be 20%-40%.^14^ Coexistence of additional lateral epicondylitis was not evaluated in our study. Patients with a diagnosis of lateral epicondylitis were excluded from the study.

There are some limitations of our study. First of all, it can be shown that it has a retrospective character, offers short-mid-term follow-ups, and does not have long-term results. The small number of our cases can also be shown among these limitations, but since it is a rare syndrome, studies in the literature have similar patient numbers.

## Conclusion

In the light of the positive clinical results we obtained, first, a correct diagnosis should be made with a careful physical examination and other differential diagnoses should be excluded. Conservative treatment including regional steroid injection as the first treatment option in diagnosed cases is absolutely necessary, and surgical release is a treatment method that we can recommend for patients who do not respond to conservative treatment for 6 months.

## Figures and Tables

**Table 1.** Demographic and Clinical Data

Number of patients		18 (7 male, 11 female)
Mean age (year)		41.5 (range = 22-61)
Involvement side		9 right and 9 left
Hand dominancy		10
Mean time to onset of symptoms		10.5 (range = 5 to 20)
Number of patients misdiagnosed as lateral epicondylitis		5
Intensity of hand use during everyday activities	None	3
	Light	4
	Moderate	6
	Vigorous	5

**Table 2. t2-eajm-55-1-59:** Clinical Outcomes of All Study Participants Treated (n = 18)

		Preoperative	Postoperative
Long finger extension test		8	3
Resisted supination test		10	5
VAS (min-max) (*P*-value)		6.38 (range = 5-8)	2.1 (range = 0-7) (*P* < .001)
q-DASH (min-max) (*P*-value)		43.4 (range = 31.8-52.5)	8.7 (range = 0-45.5) (*P* < .001)
Functional satisfaction	Very satisfied		9
	Satisfied		2
	Somewhat satisfied		
	Disappointed		2^*^
	Very disappointed		5^*^

VAS, visual analog score; q-DASH, quick-disabilities of the arm, shoulder, and hand questionnaire.

^*^Patients refractory to nonsurgical treatment who were offered surgical release.

**Table 3. t3-eajm-55-1-59:** Clinical Outcomes of Patients Refractory to Nonsurgical Treatment Who Were Offered Surgical Release

		Preoperative	Postoperative
Long finger extension test		3	1
Resisted supination test		4	1
VAS (min-max) (*P*-value)		6.1 (range: 5-7)	1,2 (range: 0-4) (p<0.001)
q-DASH (min-max) (*P*-value)		37.4 (range: 31.2-45.5)	4.7 (range: 0-13.6)
Functional satisfaction	Very satisfied		4
	Satisfied		1
	Somewhat satisfied		
	Disappointed	1^*^	1
	Very disappointed	5^*^	

VAS, visual analog score; q-DASH, Quick-Disabilities of the arm, shoulder, and hand questionnaire.

^*^Six patients who accepted surgical treatment.
